# Intracellular Environment and *agr* System Affect Colony Size Heterogeneity of *Staphylococcus aureus*

**DOI:** 10.3389/fmicb.2020.01415

**Published:** 2020-06-30

**Authors:** Nicola Häffner, Julian Bär, Vanina Dengler Haunreiter, Srikanth Mairpady Shambat, Kati Seidl, Heidi A. Crosby, Alexander R. Horswill, Annelies S. Zinkernagel

**Affiliations:** ^1^Department of Infectious Diseases and Hospital Epidemiology, University Hospital Zurich, University of Zurich, Zurich, Switzerland; ^2^Department of Immunology and Microbiology, University of Colorado School of Medicine, Aurora, CO, United States; ^3^Department of Veterans Affairs Eastern Colorado Health Care System, Denver, CO, United States

**Keywords:** *Staphylococcus aureus*, small colony variants, intracellular survival, *agr* system, intracellular pH

## Abstract

*Staphylococcus aureus* causes chronic and relapsing infections, which may be difficult to treat. So-called small colony variants (SCVs) have been associated with chronic infections and their occurrence has been shown to increase under antibiotic pressure, low pH and intracellular localization. In clinics, *S. aureus* isolated from invasive infections often show a dysfunction in the accessory gene regulator (*agr*), a major virulence regulatory system in *S. aureus*. To assess whether intracellular environment and *agr* function influence SCV formation, an infection model was established using lung epithelial cells and skin fibroblasts. This allowed analyzing intracellular survival and localization of a panel of *S. aureus* wild type strains and their isogenic *agr* knock out mutants as well as a natural dysfunctional *agr* strain by confocal laser scanning microscopy (CLSM). Furthermore, bacterial colonies were quantified after 1, 3, and 5 days of intracellular survival by time-lapse analysis to determine kinetics of colony appearance and SCV formation. Here, we show that *S. aureus* strains with an *agr* knock out predominantly resided in a neutral environment, whereas wild type strains and an *agr* complemented strain resided in an acidic environment. *S. aureus agr* mutants derived from an intracellular environment showed a higher percentage of SCVs as compared to their corresponding wild type strains. Neutralizing acidic phagolysosomes with chloroquine resulted in a significant reduction of SCVs in *S. aureus* wild type strain 6850, but not in its *agr* mutant indicating a pH dependent formation of SCVs in the wild type strain. The in-depth understanding of the interplay between intracellular persistence, *agr* function and pH should help to identify new therapeutic options facilitating the treatment of chronic *S. aureus* infections in the future.

## Introduction

*Staphylococcus aureus* is one of the major human pathogens causing both nosocomial and community-acquired infections. These may range from superficial skin and soft tissue infections to severe invasive infections including pneumonia, sepsis and endocarditis (Lowy, 1998; Petti and Fowler, 2002). Antibiotics usually effectively kill *S. aureus*. However, chronic and relapsing infections occur even if bacteria are fully susceptible to antibiotics (Welsh et al., 2011; Libraty et al., 2012; Fisher et al., 2017). One reason is the intracellular localization of *S. aureus*, which was previously considered an exclusively extracellular pathogen. Over the last decade, the relevance of intracellular bacteria as a reservoir in chronic infection has been recognized (Tuchscherr et al., 2011; Fraunholz and Sinha, 2012; Libraty et al., 2012). *S. aureus* has been shown to actively invade and survive in both professional as well as non-professional phagocytes (Sendi and Proctor, 2009; Fraunholz and Sinha, 2012; Strobel et al., 2016). Once taken up by a eukaryotic cell, the bacterium can undergo different fates: (i) it escapes the phagosome, proliferates in the cytosol and eventually kills its host cell, (ii) it is killed by the host’s defense machinery or (iii) it stays intracellularly without being cleared (Fraunholz and Sinha, 2012). Intracellular localization is a niche for *S. aureus*, where it is protected from the host’s immune system as well as from antibiotics that act extracellularly. Intracellular *S. aureus* has been described in cases of recurrent infections such as osteomyelitis and rhinosinusitis (Bosse et al., 2005; Clement et al., 2005; Libraty et al., 2012). Bacteria recovered from chronic infections often show a heterogeneity in colony size (Proctor et al., 2006). The smaller colonies represent a bacterial subpopulation that has previously been referred to as small colony variants (SCVs) (Proctor et al., 1994). Recently, our group showed that one reason for a SCV subpopulation is a delayed growth resumption imposed by a stress such as acidic pH, antibiotic exposure or the intracellular milieu of eukaryotic host cells (Leimer et al., 2016; Vulin et al., 2018). In particular, prolonged intracellular survival of *S. aureus* has been linked to an increasing percentage of SCVs in a bacterial population (Tuchscherr et al., 2011; Leimer et al., 2016; Rollin et al., 2017). In addition to these extrinsic factors, genetic factors including several *S. aureus* regulatory systems such as *agr*, *sigB* and *sarA* were shown to influence SCV formation (Kahl et al., 2005). Especially, the accessory gene regulator (*agr*), a quorum sensing system regulating the expression of adhesins during attachment and virulence factors during infection, is downregulated in SCVs (Moisan et al., 2006; Mitchell et al., 2013). The majority of *S. aureus* isolated from invasive infections showed reduced toxicity accompanied by a reduction in *agr* activity as compared to colonizing *S. aureus* strains (Laabei et al., 2015; Altman et al., 2018). Downregulation of *agr* has been reported to promote bacteremia and increase mortality in invasive *S. aureus* infections (Shopsin et al., 2008; DeLeo et al., 2011; Schweizer et al., 2011; Soong et al., 2015). The role of *agr* deficiency together with the exposure to intracellular pH on SCV formation has not been elucidated yet.

In this study, we show that despite their localization in a neutral intracellular milieu, *S. aureus agr* mutants have an increased colony size heterogeneity as compared to their corresponding wild type strains suggesting a pH independent formation of SCVs in *S. aureus agr* mutants.

## Materials and Methods

### Cell Culture, Bacterial Strains and Growth Conditions

Human alveolar lung epithelial cells A549 and human skin fibroblast cells BJ-5ta were cultured in Dulbecco’s Modified Eagle Medium (DMEM) (Gibco) supplemented with 4.5 g/L glucose, 2 mM L-glutamine (Gibco) and 10% fetal bovine serum (FBS) (Eurobio) at 37°C and 5% CO_2_. All *S. aureus* strains used in this study are listed in Table 1. *S. aureus* overnight cultures were grown in tryptic soy broth (TSB) (BD) at 37°C, 220 rpm. Prior to infection, bacterial overnight cultures were diluted 1:10 in TSB and grown for 2 h to exponential phase. Phage 11 was used to transduce an existing *agr:tetM* mutation from *S. aureus* strain RN6911 into strain 6850 (Novick et al., 1993). All *agr* mutant strains used in this study were whole genome sequenced using an Illumina MiSeq machine and the QIAseq FX DNA Library Kit (Qiagen) to confirm correct replacement of the whole *agr* system by *tetM*. Whole genome data analysis was performed using CLC Genomic Workbench (Qiagen) version 10.1.1.

**TABLE 1:** *S. aureus* wild type and *agr* mutant strains used in this study.

Strain	Characteristics	References
RN6911	*agr* has been replaced with the tetracycline resistance gene (*tetM*)	Novick et al., 1993
Cowan I	obtained from a patient with septic arthritis	ATCC 12598
	naturally non-functional Agr system	Bohacek et al., 1971
SH1000 wt	functional rsbU derivative of strain 8325-4	Horsburgh et al., 2002
SH1000 *agr ^–^*	SH1000 *agr*:*tetM*, Tc^r^	Horsburgh et al., 2002
6850 wt	obtained from a patient with osteomyelitis	ATCC 53657
		Balwit et al., 1994
6850 *agr ^–^*	6850 *agr*:*tetM*, Tc^r^	This study
JE2 wt	plasmid-cured derivative of strain LAC	NARSA
JE2 *agr ^–^*	JE2 *agr*:*tetM*, Tc^r^	Seidl et al., 2017
LAC **ϕ** agr	LAC *agr*:*tetM* complemented with ϕ11:LL29_RNAIII*agrBDCA*, Tc^r^	AH5396, This study
SH1000 **ϕ** agr	SH1000 *agr*:*tetM* complemented with ϕ11:LL29_RNAIIIagrBDCA, Tc^r^	This study

*Tc^*r*^, tetracycline resistant.*

To construct the *agr* complemented strain, RNAIII and the *agrBDCA* operon were amplified from USA300 LAC genomic DNA using primers HC735 (5′-GTTGTT GGATCC ACGGCGGGTCTCATAATGAT-3′) and HC736 (5′-GTTGTT AAGCTT TGCGCCATAGGATTGTAGAGT-3′). The PCR product was digested using *Bam*HI and *Hin*dIII, and ligated into pLL29 containing the tetracycline resistance gene *tetK* (Luong and Lee, 2007), generating plasmid pHC175. This plasmid was electroporated into *S. aureus* 4220 containing pLL2787, and chromosomal integration of plasmid pHC175 at the phage 11 attachment site was selected on TSA containing 1 μg/ml tetracycline. The integrated cassette was transduced into *S. aureus* LAC using phage 11, and integration was confirmed by PCR. The Δ*agr*:*tetM* deletion was transduced into this strain, selecting on TSA containing 0.2 μg/ml minocycline. The *agr* deletion was confirmed by PCR and phenotypes were verified on blood plates. Phage 11 was used to transduce the construct for *agr* complementation into *S. aureus* SH1000 *agr*:*tetM.*

### Bacterial Infection and Intracellular Survival Assay

For all assays, 10^5^ epithelial cells were seeded into 24-well plates. For confocal microscopy, 12 mm coverslips were placed inside the wells before seeding. Epithelial cells were infected at a multiplicity of infection (MOI) of 1 for intracellular survival, microscopy and radial colony growth analysis. After 3 h invasion, epithelial cells were treated with 1 mg/ml flucloxacillin and 20 μg/ml lysostaphin for 1 h (D0), 1 (D1), 3 (D3), or 5 days (D5), respectively. To neutralize the phagolysosomes, 20 μM chloroquine was additionally added. Medium was changed daily. To assess bacterial intracellular survival at the indicated time points, epithelial cells were washed twice with phosphate buffered saline (PBS) (Gibco) and lysed with water. Colony forming units (CFUs) were recovered on tryptic soy agar (TSA) plates.

### Radial Colony Growth and SCV Formation

Intracellular *S. aureus* was recovered 4 h, 1, 3, and 5 days after infection as described above. Radial colony growth on blood agar plates (Columbia + 5% sheep blood, Biomerieux) was monitored at 37°C. For time-lapse imaging, four cameras (Basler, acA5472-17um 20MP with Fujinon Objectives CF16ZA-1S 16 mm/1.8M37.5 × 0.5) were used and images acquired every 10 min for 48 h. Cameras were triggered by Basler’s pylon software suite (Version: 6.0.1). For single time points imaging after 24 h of growth on agar plates, a Canon EOS 1200D reflex camera was used. All images were analyzed with ColTapp, an automated image analysis application for efficient microbial colony growth dynamics quantification (doi: https://doi.org/10.1101/2020.05.27.119040). Appearance time was estimated as the time when a colony reached a radius of 200 μm. Colony growth delay after 1, 3, and 5 days of infection was defined as the difference of the appearance time of a colony compared to the mean appearance time after 4 h infection (day 0). Similarly, radius deviation was defined as the difference in radius of a colony compared to the mean radius at the 4 h infection time point. A colony was defined as a SCV if the area of a colony after 24 h regrowth was smaller than 1/5th of the median area of a colony of the 4 h infection time point after 24 h regrowth (Vulin et al., 2018). A total of 9,693 colonies were analyzed with automated time-lapse analysis and 23,716 colonies with single time point imaging of plates. Plates with less than 15 and more than 350 colonies were excludes in further analyses.

### Confocal Microscopy

Fluorescent images of intracellular bacteria were taken with the confocal laser scanning microscope (CLSM) Leica SP8 inverse. Samples were fixed at the indicated time points in 4% paraformaldehyde (PFA). Eukaryotic nuclei and bacterial DNA were stained with Hoechst 33342 (Thermo Scientific), late endosomes and lysosomes were stained with 1 μg/ml mouse α-lysosome associated membrane protein 2 (Lamp-2) (BD Pharmigen) and *S. aureus* was stained with 2 μg/ml rabbit α-*Staphylococcus aureus* (EastCoast). As secondary antibodies, 2 μg/ml goat α-mouse Alexa 647 (Invitrogen) and 2 μg/ml goat α-rabbit Alexa 488 (Abcam) were used to label Lamp2 and *S. aureus*, respectively. To identify acidic compartments, 1 μM LysoTracker Red DND-99 (Invitrogen) was used. Images were deconvolved with Huygens Professional software and further processed with the image analysis software Imaris.

### Flow Cytometry-Based pH Monitoring

To monitor intracellular pH, we adapted the method established by Sinha et al. (1999). Briefly, bacterial overnight cultures were pelleted and resuspended in sodium bicarbonate buffer, pH 9. *S. aureus* strains were labeled with 100 μg/ml fluorescein isothiocyanate (FITC) for 1 h at 37°C, shaking. Epithelial cells were infected at a MOI of 25 for 3 h, followed by 1, 3, or 5 days treatment with 1 mg/ml flucloxacillin and 20 μg/ml lysostaphin. Prior to flow cytometry, epithelial cells were trypsinized, pelleted and resuspended in PBS with 1:500 diluted aqua fluorescent reactive dye (Invitrogen) and 1 μM carbonyl cyanide m-chlorophenyl hydrazone (CCCP) for 15 min at 37°C. Quenching of FITC signal corresponded to an acidic pH. Total internalized bacteria treated with CCCP were calculated according to the formula: AFU = (mean fluorescence intensity BL1-A × percentage of cells in Q3)/1,000. Q3 is the quadrant negative for aqua, but positive for FITC signal. The difference in arbitrary fluorescence units (AFU) between samples treated with CCCP and untreated was calculated as an indirect readout for intracellular pH using the formula: ΔAFU=(AFU_−CCCP_-*AFU*_+ CCCP_)/(AFU_+ CCCP_× 10,000). Values close to zero indicated neutral pH and larger negative values indicated acidic pH. Positive values were considered as below detection limit and set to zero in the analysis. Samples were measured with the Attune NxT Acoustic Focusing Cytometer (Thermo Fisher Scientific) and analyzed using the software FlowJo.

### Cell Viability Assay

To assess bacterial cytotoxicity, a MTT assay was performed, as previously described (Seidl and Zinkernagel, 2013). Epithelial cells were infected at a MOI of 1 as described above. One hour, 1, 3, and 5 days after invasion, the tetrazolium dye MTT (3-[4,5-dimethylthiazole-2-yl]-2,5-diphenyltetrazolium bromide) was added directly to the medium at a final concentration of 1 mg/ml and incubated for 2 h at 37°C and 5% CO_2_. Medium was aspirated and formazan crystals were dissolved in 0.04 M HCl in isopropanol. Absorbance was measured at 570 nm with the VERSAmax tunable microplate reader (Molecular Devices).

### Lactate Dehydrogenase (LDH) Release

To monitor lysis of eukaryotic cells, epithelial cells were infected at a MOI of 1 as described above. Three hours and 24 h after infection cell supernatants were collected. Eukaryotic cell lysis was quantified using the CytoTox 96^®^ Non-Radioactive Cytotoxicity Assay (Promega). Briefly, an equal volume of CytoTox 96^®^ Reagent was added to the supernatant. After 30 min, the Stop Solution was added and absorbance was measured at 490 nm.

### Statistical Analysis

All statistical analysis were conducted with R and R Studio Version 3.5.1 (R Core Team, 2018). Figures were produced with ggplot (Wickham, 2016) and ggbeeswarm (Clarke and Sherrill-Mix, 2017), pairwise comparisons were calculated based on estimated marginal means (emm) (Russell, 2018) and p-values were corrected for multiple testing based on Tukey’s or Sidak’s method. We separated the analysis of *S. aureus* strain Cowan from the analysis of the three wild type and *agr* mutant pairs. CFU/ml was log_10_-transformed and linear regression was used to assess the effect of the duration of infection for strain Cowan separately. For the other wild type and *agr* mutant strains a model incorporating the interaction between days of infection, strain and presence or absence of *agr* was additionally used. Following, emm pairwise comparisons between wild types and *agr* mutants averaging over time were calculated per strain. Number of SCV versus normal sized colonies per replicate were modeled with a generalized linear model (glm) based on a quasi-binomial distribution to account for overdispersion. We included the three-way interaction term between days of infection, strain and presence or absence of *agr* as well as all possible two-way interactions and single parameter effects. Emm pairwise differences were calculated to compare wild types and *agr* mutants per strain and for each day. Additionally, we investigated the effect of chloroquine treatment at day five (the untreated data was used in two separate analysis). Similarly, a glm with a quasi-binomial distribution and a three-way interaction term including sub-interactions of chloroquine treatment, strain and presence or absence of *agr* was used. Pairwise differences were calculated to compare wild types and *agr* mutants per strain and within chloroquine treatment. For strain Cowan, a simple glm with duration of infection as the only term was used. A linear regression model with the three-way interaction term between the three strains, presence or absence of *agr* and chloroquine treatment was used to analyze pH assay data at day five. Emm pairwise differences were calculated to compare wild types and *agr* mutants per strain and within chloroquine treatment. For strain Cowan, a simple linear regression with duration of infection as the only term was used.

## Results

### *S. aureus* Cowan Resides in an Acidic Environment Over a Period of Five Days

**FIGURE 1:**
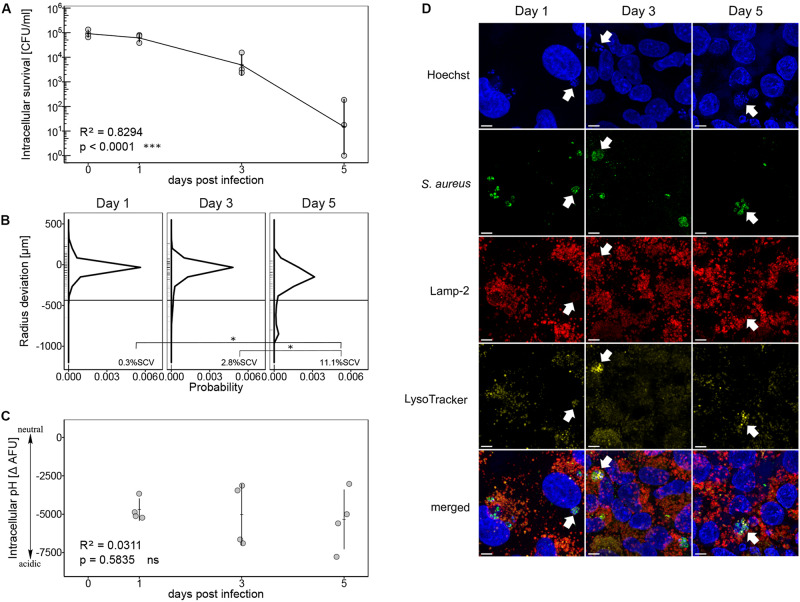
Intracellular survival of *S. aureus* strain Cowan in acidic endosomes. **(A)** Recovered bacteria are shown as CFU per ml in log scale over an infection period of 5 days. Three independent experiments in technical triplicates ± SEM are shown. **(B)** Colony size distribution of recovered bacteria. After 24 h regrowth, colony radius was normalized to the mean colony area at day 0 (Supplementary Figure 1). Radius size deviation from reference is plotted as probability density function. Individual colonies are shown as short lines on y-axis. The threshold for SCVs, indicated as a horizontal line, was calculated as 1/5th of the area of the median area at day 0. The mean percentage of SCVs is written in each panel. Colonies from single images as well as from time-lapse analysis were taken into account. For day one 607 colonies, for day three 362 colonies and for day five 84 colonies were analyzed in two to four biological repeats. Pairwise comparison of day one and day five ^∗^*p* = 0.0172, pairwise comparison of day three and day five ^∗^*p* = 0.0362. **(C)** Measurement of intracellular pH after 1, 3, and 5 days of infection. Four independent experiments in technical triplicates ± SEM are shown. **(D)** CLSM of intracellular *S. aureus* strain Cowan. Co-localization of *S. aureus* strain Cowan with Lamp-2-positive compartments and LysoTracker signal are indicated by arrows. Length of scale bar is 5 μm.

In a first step, we monitored intracellular survival of *S. aureus* strain Cowan over a period of 5 days within human lung epithelial cells A549. Throughout the infection, the number of intracellular bacteria significantly decreased (*p* < 0.0001). After 5 days, surviving bacteria decreased by four log, however, intracellular bacteria were not completely eradicated (Figure 1A). Bacteria recovered after 5 days showed a broad colony size heterogeneity (Figure 1B). The probability density function showed a peak of colony size deviation from the mean colony size at day 0 (Supplementary Figure 1) indicating a mean colony size reduction of 20 to 35 μm after 1 and 3 days intracellular survival with 0.3% ± 0.3 SEM and 3.7% ± 1.7 SEM SCVs, respectively. After 5 days, the colony size distribution became broader with a mean colony size reduction of 200 μm. We observed a significant increase in SCV formation from day one to day five (*p* = 0.017) as well as from day three to day five (*p* = 0.036) with 11% ± 0.7 SEM SCVs on day five (Figure 1B). The threshold for SCVs was set at 1/5th of the median colony area at day 0. To investigate whether colony size heterogeneity was linked to the exposure of acidic intracellular pH, we used pH dependent quenching of FITC-labeled bacteria as described by Sinha et al. (1999). Throughout the infection, low ΔAFU values were measured indicating exposure of *S. aureus* strain Cowan to an acidic environment (Figure 1C). Linear regression showed no evidence of a changing intracellular pH over time (*p* = 0.5835). Intracellular localization of surviving bacteria in an acidic compartment was additionally confirmed by confocal laser scanning microscopy (CLSM). Co-localization of bacteria with lysosome-associated membrane protein 2 (Lamp-2) and LysoTracker signal indicated the localization of bacteria in acidic endosomes (Figure 1D).

### Intracellular Survival of *S. aureus* Wild Type Strains and Their Corresponding *agr* Mutants

**FIGURE 2:**
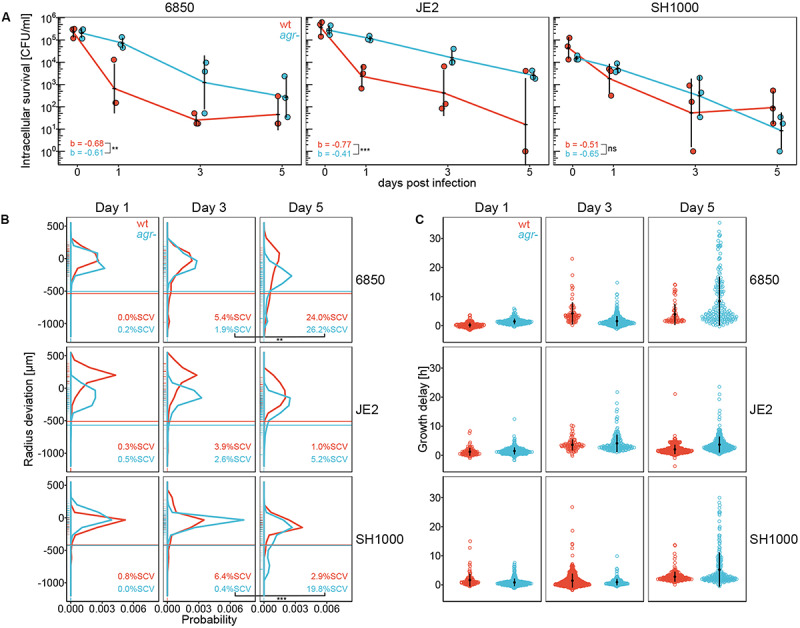
Intracellular survival, colony size distribution and lag time of *S. aureus* wild type strains (wt) and *agr* mutants (*agr-*). *S. aureus* wild type strains are indicated in red, *agr* mutants are indicated in blue. **(A)** Intracellular survival of *S. aureus* wild type strains and *agr* mutants in A549 cells. Recovered bacteria are shown as CFU per ml in log scale over an infection period of 5 days. Data points of wild types and *agr* mutants are offset on x-axis for easier readability. Three independent experiments in technical triplicates ± SEM are shown. **p = 0.0032, ***p = 0.0005. b indicates the slope of the curve. **(B)** Colony size distribution of recovered bacteria. After 24 h regrowth, colony radius is normalized to the strain specific mean colony area at day 0 (Supplementary Figure 1). Radius size deviation from reference is plotted as probability density function. Individual colonies are shown as short lines on y-axis. The threshold for SCVs, indicated as a horizontal line, was calculated as 1/5th of the area of the median area at day 0. The mean percentage of SCVs is written in each panel. Colonies from single images as well as from time-lapse analysis were taken into account. In total 11,926 colonies in two to seven biological repeats were analyzed. **p = 0.0024, ***p < 0.0001. **(C)** A subset of the colonies shown in B were monitored with time-lapse imaging on blood agar plates. Appearance time is normalized to the strain specific mean appearance time at day 0 (Supplementary Figure 7). Growth delay is plotted as deviation from reference in hours. Every point of the dot plot represents one colony. In total 3,715 colonies in two to four biological repeats were analyzed. Colony radius at 24 h of time-lapse analysis is included in Figure 2B.

*S. aureus* wild type strain Cowan shows a natural *agr* defective phenotype (Grundmeier et al., 2010) and resides in acidic compartments inside epithelial cells (Figure 1D). To evaluate whether *agr* deficiency or the survival inside acidic compartments contributed to the formation of SCVs, we analyzed a panel of *S. aureus* wild type strains and their isogenic *agr* knock out mutants (Table 1). *S. aureus* strain 6850 was included in this study because it has previously been shown to increase intracellular pH after 6 h of infection of HeLa cells and of human embryonic kidney cells 293 (Lam et al., 2010). JE2 is a highly virulent *S. aureus* strain representative of the methicillin-resistant USA300 lineage (Tenover and Goering, 2009) and SH1000 is a commonly used laboratory strain of the 8325 lineage (Horsburgh et al., 2002; O’Neill, 2010). Wild type strains and their *agr* mutants were tested for their ability to invade and reside inside human lung epithelial cells A549. *S. aureus* CFUs were recovered from eukaryotic cells 1, 3, and 5 days after infection. A significant decrease in intracellular survival over time was observed for all *S. aureus* strains (*p* < 0.0001) (Figure 2A). We further performed pairwise comparisons of *S. aureus* wild types and *agr* mutants and found that *S. aureus* wild type strains JE2 and 6850 showed a significant decrease in intracellular survival as compared to their corresponding *agr* mutants (*p* = 0.0005 and *p* = 0.0032, respectively). Although bacterial numbers recovered from epithelial cells decreased over time, neither *agr* mutants nor wild type strains were eradicated completely. Additionally, we monitored eukaryotic cell survival and cytotoxicity. *S. aureus* wild type strains showed a higher cytotoxicity as compared to their corresponding *agr* mutants. Despite the initial cell loss caused by *S. aureus* wild type strains within the first 24 h (Supplementary Figure 2A), eukaryotic cells showed recovery after 3 and 5 days (Supplementary Figure 2B).

### Increased SCVs Due to a Delay in Colony Appearance After Intracellular Survival

The influence of intracellular survival on colony size heterogeneity was determined by quantifying colony radius on blood agar plates after 1, 3, and 5 days of infection. After 3 days of intracellular survival, 6850, JE2 and SH1000 wild type strains showed a higher percentage of SCVs as compared to their *agr* mutants (Figure 2B). This phenotype switched after 5 days of intracellular survival. All *S. aureus agr* mutants showed a broader colony size heterogeneity with higher percentage of SCVs as compared to their corresponding wild type strains 5 days after infection (Figure 2B and Supplementary Figure 3, left panel). Pairwise comparisons of wild types and *agr* mutants over time showed a significant increase in the percentage of SCVs in 6850 and SH1000 *agr* mutant strains from day three to day five (*p* = 0.0024 and *p* < 0.0001, respectively). Similar to the cell line A549 results, we observed a trend toward smaller colony sizes in *S. aureus agr* mutants as compared to *S. aureus* wild type strains after 5 days intracellular exposure in the fibroblast cell line BJ5-ta (Supplementary Figures 4A, 5A). Similar to SH1000 wild type strain, complementation of the SH1000 *agr* mutant resulted in lower SCV numbers as compared to the SH1000 *agr* mutant (Supplementary Figures 5B, 6C). We further investigated whether smaller colonies were linked to a delay in colony appearance as previously described using automated colony time-lapse analysis (Vulin et al., 2018). To observe differences in appearance time, radial colony growth was monitored and normalized to the mean appearance time at day 0 (Supplementary Figure 7). After 3 days of infection, *S. aureus* wild type strains 6850 and SH1000 showed more late appearing colonies as compared to their *agr* mutants, whereas the JE2 *agr* mutant showed more late appearing colonies than the JE2 wild type (Figure 2C). Similar to the phenotype switch observed in SCV formation from day three to day five, we observed more late appearing colonies for all *agr* mutants as compared to their corresponding wild type strains after 5 days of infection (Figure 2C). Based on these findings, we conclude that SCVs are linked to the delay in colony appearance.

### Acidic Intracellular Milieu Affects Colony Size Heterogeneity of *S. aureus* Wild Type Strains

**FIGURE 3:**
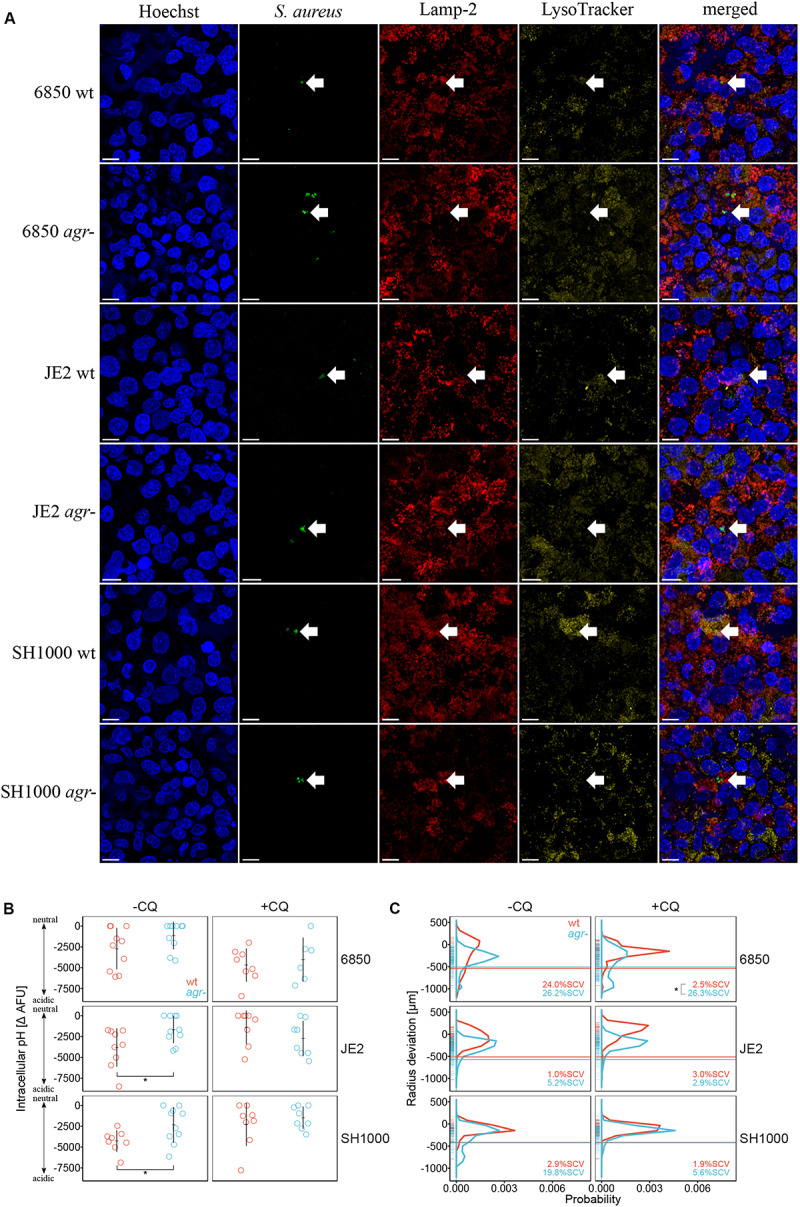
Acidic intracellular pH triggers SCV formation in *S. aureus* wild type strains. *S. aureus* wild type strains (wt) are indicated in red, *agr* mutants (*agr*-) are indicated in blue. **(A)** Intracellular localization of *S. aureus* strains 5 days after infection shown by arrows. Co-localization of *S. aureus* wild type strains with Lamp-2 and LysoTracker indicate the localization in acidic endosomes. Length of scale bar is 10 μm. **(B)** Measurement of intracellular pH after 5 days of infection. The alkalizing agent chloroquine (CQ) was used to neutralize phagolysosomes. −CQ indicates no treatment, +CQ indicates chloroquine treatment. Eight to ten independent experiments in technical triplicates ± SEM are shown. Pairwise comparison of JE2 wt and *agr-* *p = 0.0243, pairwise comparison of SH1000 wt and *agr-* *p = 0.0486. **(C)** Comparison of colony size distribution of untreated (−CQ) and chloroquine treated (+CQ) bacteria recovered after 5 days of infection. After 24 h regrowth, colony radius is normalized to the strain specific mean colony area at day 0 (Supplementary Figure 1). Radius size deviation from reference is plotted as probability. The threshold for SCVs, indicated as a horizontal line, was calculated as 1/5th of the area of the median area at day 0. The mean percentage of SCVs is written in each panel. Colonies from single images as well as from time-lapse analysis were taken into account. Colony radius deviation of untreated bacteria is also shown in Figure 2B. In total 9,381 colonies (4,387 untreated and 4,994 chloroquine treated) in two to five biological repeats were analyzed. *p = 0.0209.

To elucidate the correlation between intracellular pH and *agr* deficiency, intracellular localization of *S. aureus* wild type strains and *agr* mutants was investigated by CLSM using LysoTracker to stain acidic compartments. We observed co-localization of LysoTracker signal with *S. aureus* wild type strains indicating their localization in acidic compartments, but no co-localization of LysoTracker with their respective *agr* mutants was identified (Figure 3A). We further quantified intracellular pH of infected eukaryotic cells by measuring quenching of FITC-labeled bacteria. Based on a three-way interaction model, we found that *S. aureus* wild type strains SH1000 and JE2 were exposed to a significantly lower intracellular pH represented as larger negative ΔAFU values as compared to their corresponding *agr* mutants (*p* = 0.049 and 0.024, respectively) 5 days after infection (Figure 3B). The same trend was observed for the 6850 wild type strain as compared to its *agr* mutant (*p* = 0.091). Similar to our observations in the cell line A549 we observed that *S. aureus* wild type strains resided in a more acidic intracellular environment as compared to their corresponding *agr* mutants in the fibroblast cell line BJ5-ta (Supplementary Figure 4B). Complementation of the SH1000 *agr* mutant resulted in exposure to lower pH, comparable to the ΔAFU values obtained from SH1000 wild type strain (Supplementary Figure 6D). The weak base chloroquine was used as an alkalizing agent to neutralize phagolysosomes. After 5 days of chloroquine treatment there was no significant difference in pH between wild type strains and *agr* mutants (Figure 3B). ΔAFU values for day one and day three are shown in Supplementary Figure 8. To assess whether intracellular pH influenced colony size heterogeneity and the formation of SCVs in *S. aureus*, colony radius was measured after 5 days of chloroquine treatment (Figure 3C). Percentage of SCVs and colony size distribution for day one and three after chloroquine treatment are shown in Supplementary Figure 3 (right panel) and Supplementary Figure 9, respectively. Addition of the alkalizing agent chloroquine resulted in significant decrease in percentage of SCVs in *S. aureus* wild type strain 6850 as compared to its *agr* mutant (*p* = 0.0209) after 5 days of infection (Figure 3C). An up to three-fold decrease in the percentage of SCVs was observed in *S. aureus* strains SH1000 and JE2 after chloroquine treatment.

## Discussion

We found that *S. aureus agr* knock out strains showed increased intracellular survival and higher SCV numbers after recovery from intracellular exposure as compared to *S. aureus* wild type strains. Colony size heterogeneity of *agr* mutants was independent from intracellular pH, whereas an acidic intracellular pH was necessary to trigger SCV formation in wild type strains.

*S. aureus* wild type strains JE2 and 6850 showed a significant decrease in intracellular survival as compared to their corresponding *agr* mutants. It has previously been shown that an isogenic *agr* mutant of JE2 survives at higher rates inside HeLa cells for 24 h as compared to JE2 wild type strain (Munzenmayer et al., 2016). To our knowledge, the effect of prolonged intracellular exposure and its influence on colony size heterogeneity leading to SCV formation in a panel of *S. aureus* wild type strains and their respective isogenic *agr* mutants has not been studied in detail so far. A functional *agr* facilitates bacteria to express phenol soluble modulins (PSMs) among which PSMα acts as a pore-forming toxin to promote escape from endosomes (Schnaith et al., 2007; Giese et al., 2011; Grosz et al., 2014; Strobel et al., 2016). Once in the cytosol, the bacterium is able to multiply and eventually disrupt the host cell (Fraunholz and Sinha, 2012). We and others (Haslinger-Löffler et al., 2006; Strobel et al., 2016; Seidl et al., 2017) previously showed that *S. aureus* strains JE2 and 6850 are more cytotoxic than Cowan. In general, bacterial cytotoxicity is highly MOI-dependent. As shown by the LDH release assay, JE2 and 6850 efficiently lysed the eukaryotic cells and thus escaped the host cell reaching the medium containing lysostaphin and flucloxacillin, which killed the bacteria. This explains the drastic decrease in CFU counts for *S. aureus* wild type strains JE2 and 6850. Despite the different rates of CFU reduction, a small population of both *S. aureus* wild types and *agr* mutants resided intracellularly up to 5 days after infection. Tuchscherr and colleagues observed intracellular survival of *S. aureus* strain 6850 even after 28 days of infection of A549 cells (Tuchscherr et al., 2011). The phenotypically *agr* defective *S. aureus* strain Cowan showed comparable survival rates to the *agr* mutants of JE2 and 6850. However, *S. aureus* wild type strain SH1000 showed similar intracellular survival as its *agr* mutant. SH1000 carries a 63 bp deletion in the *spa-sarS* intergenic region that leads to a 75% decrease in the SarS activator steady state level, which is known to influence *agr* activity (Cheung et al., 2001; Blevins et al., 2002; Oscarsson et al., 2006; Baek et al., 2013). Proteomics analysis of *S. aureus* strain SH1000 by Strobel and colleagues revealed low levels of hemolysins, PSMs and other toxins under *agr* control, suggesting a reduced *agr* activity in SH1000 as compared to other *S. aureus* wild type strains including 6850 and a USA300 strain (Strobel et al., 2016). The reduced *agr* activity is a potential explanation for the similarities between SH1000 wild type and its *agr* mutant regarding intracellular survival. A review by Löffler et al. (2014) summarizes *S. aureus* intracellular survival inside different cell lines. They conclude that intracellular localization imposes a selective pressure for slow growing bacteria referred to as SCVs. Throughout the infection, we observed increasing colony size heterogeneity in all *S. aureus* strains tested. After 3 days of infection, *S. aureus* wild type strains 6850 and SH1000 showed a higher percentage of SCVs as compared to their *agr* mutants. We investigated colony size heterogeneity in more detail by analyzing colony’s first appearance on blood agar plates and found that the SCV numbers reflected the number of late appearing colonies. Our group previously showed that small colony sizes are indirectly proportional to the delay in colony appearance (Vulin et al., 2018). However, after 5 days of infection, this phenotype switched as *agr* mutants of JE2, 6850 and SH1000 showed an even broader colony size distribution with higher percentage of SCVs as compared to their corresponding wild type strains. The earlier onset of SCV formation observed for *S. aureus* wild type strains might be due to the additional pressure of residing in an acidic intracellular environment as compared to *S. aureus agr* mutants, which were exposed to a less acidic environment. We showed that 6850 and SH1000 wild type strains were exposed to a slightly more acidic intracellular environment as compared to their respective *agr* mutants already after 1 and 3 days of intracellular survival. Therefore, *S. aureus* wild type strains were exposed to an acidic milieu for the whole infection period of 5 days. Additionally, CFU numbers of recovered *S. aureus* wild type strains showed a three log reduction after 3 days of intracellular survival whereas CFU numbers of *S. aureus agr* mutants only decreased by one log. High numbers of normally growing colonies might mask the population of SCVs. This possibly explains the higher proportion of SCVs in *S. aureus* wild type strains as compared to their corresponding *agr* mutants at day three. The factors and mechanism causing this switch in SCV numbers between day three and day five need to be further studied in detail. The increase in SCV formation in *S. aureus agr* mutants was also reflected by an increase of late appearing colonies 5 days after infection. Intracellular survival and exposure to acidic pH have been shown to trigger the formation of SCVs (Tuchscherr et al., 2011; Leimer et al., 2016; Vulin et al., 2018). Endosomal maturation is characterized by a drop in pH (Maxfield and Yamashiro, 1987; Huotari and Helenius, 2011), therefore phagocytosed bacteria are likely exposed to an acidic pH inside late endosomes. To check whether acidic pH or dysfunctional *agr* triggers SCV formation, we monitored intracellular pH of *S. aureus* wild type strains and their *agr* mutants. We found that JE2 and SH1000 wild type strains were exposed to significantly lower intracellular pH as compared to their corresponding *agr* mutants 5 days after infection. The same trend toward exposure to more acidic environment was also observed for 6850 wild type strain as compared to its *agr* mutant. In this study, Cowan is the only strain with a defective *agr* system that resided in an acidic compartment over the entire infection period of 5 days. In contrast to the *agr* mutants of 6850, JE2 and SH1000, in which the complete *agr* system is knocked out and replaced with a tetracycline resistance cassette, the *agr* system of Cowan is still present in the genome. It was shown by Grundmeier et al. (2010), that Cowan expresses lower levels of *hla* and *agrA* as compared to the *S. aureus* strain 6850 suggesting an *agr* dysfunction. Nevertheless, baseline levels of *agr* expression might be sufficient to observe exposure to low intracellular pH comparable to other *S. aureus* wild type strains. The exposure to acidic intracellular pH resulted in larger colony size heterogeneity and an increased percentage of SCVs. The weak base chloroquine, routinely used for treatment of malaria and rheumatic diseases, was used to alkalize acidic phagolysosomes (Leimer et al., 2016). Alkalization resulted in a nine-fold reduction of SCVs in *S. aureus* wild type strain 6850, but no reduction in percentage of SCVs was observed in its corresponding *agr* mutant. Since *S. aureus agr* mutants were already exposed to a neutral environment inside the host cell, the neutralizing effect of chloroquine on SCV formation was not as strong as compared to the effect on SCV formation in wild type strains. We conclude that an acidic intracellular environment facilitates SCV formation in *S. aureus* wild type strains. In contrast, *agr* mutants are able to form high numbers of SCVs in a pH independent manner. These results suggest that a lack of *agr* regulation promotes the formation of SCVs in the intracellular environment. It was shown by Arvidson and Holme (1971) and by Weinrick et al. (2004) that post-exponential induction of *agr-*dependent genes is reduced in a nutrient rich media buffered to acidic pH. Therefore, the acidic intracellular milieu and the intracellular environment that *S. aureus* wild type strains are exposed to, might affect the regulation of the *agr* system, which in turn could contribute toward the formation of SCVs.

Our findings give more insights into the interplay between intracellular survival, *agr* function and intracellular pH contributing toward SCV formation. This will allow designing new therapeutic strategies aiming to improve therapy of chronic *S. aureus* infections in the future.

## Data Availability Statement

The datasets generated for this study are available on request to the corresponding author.

## Author Contributions

AZ, SM, and NH conceived and planned the experiments. NH carried out the experiments. KS created *agr* knockout mutants in *S. aureus* strains JE2 and 6850. HC and AH created the construct for *agr* complementation and revised the manuscript. JB analyzed the time-lapse data, did the statistical analysis and revised the manuscript. NH, VD, SM, and AZ wrote and revised the manuscript. All authors contributed to the article and approved the submitted version.

## Conflict of Interest

The authors declare that the research was conducted in the absence of any commercial or financial relationships that could be construed as a potential conflict of interest.
