# Supplementary material for: Intracellular Environment and agr System Affect Colony Size Heterogeneity of Staphylococcus aureus
Source: Front Microbiol. 2020 Jun 30;11:1415. doi: 10.3389/fmicb.2020.01415 (PMC7339952; doi:10.3389/fmicb.2020.01415)
Supplement: Supplementary file 1 [file Data_Sheet_1.pdf]

Supplementary Information

Intracellular environment and *agr* system affect colony size heterogeneity of *Staphylococcus aureus*

Nicola Häffner[1], Julian Bär[1], Vanina Dengler Haunreiter[1], Srikanth Mairpady Shambat[1], Kati Seidl[1], Heidi A. Crosby[2], Alexander R. Horswill[2,3], Annelies S. Zinkernagel[1]*

1 Department of Infectious Diseases and Hospital Epidemiology, University Hospital Zurich, University of Zurich, 8091, Zurich, Switzerland

2 Department of Immunology and Microbiology, University of Colorado School of Medicine, Aurora, Colorado, USA

3 Department of Veterans Affairs Eastern Colorado Health Care System, Denver, Colorado, USA

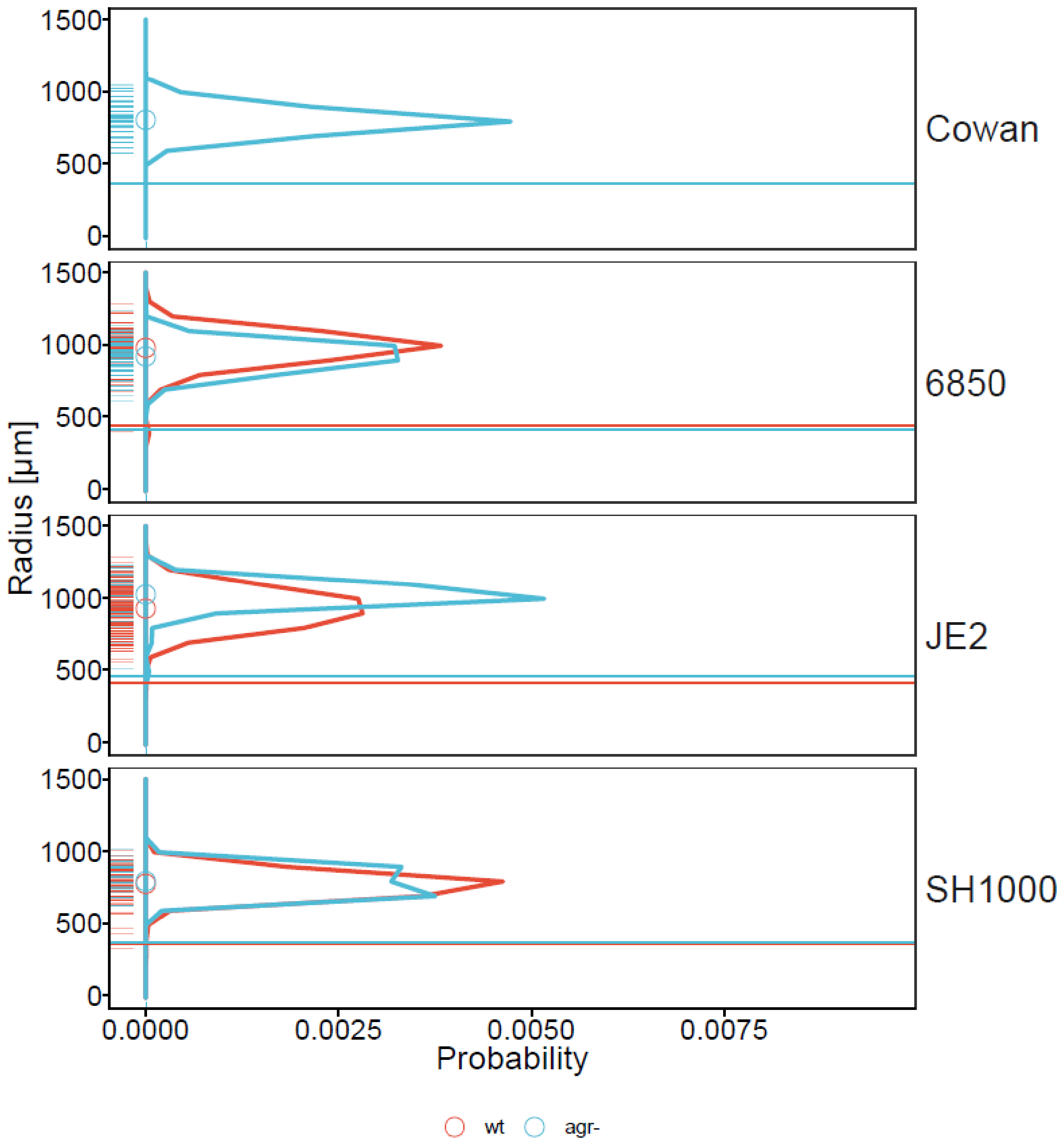

Supplementary Figure 1. Colony size distribution at day 0. After 24 h regrowth on blood agar plates, colony radius was measured. Radius size is plotted as probability density function. Individual colonies are shown as short lines on y-axis. Long horizontal lines indicate the thresholds for SCVs calculated as 1/5th of the area of the median area for each strain. Circles on y-axis indicate mean radius used as reference. Colonies from single images as well as from time-lapse analysis were taken into account. For Cowan 782 colonies, for 6850 1,381 colonies, for JE2 1,954 colonies and for SH1000 989 colonies were analyzed in four biological repeats.

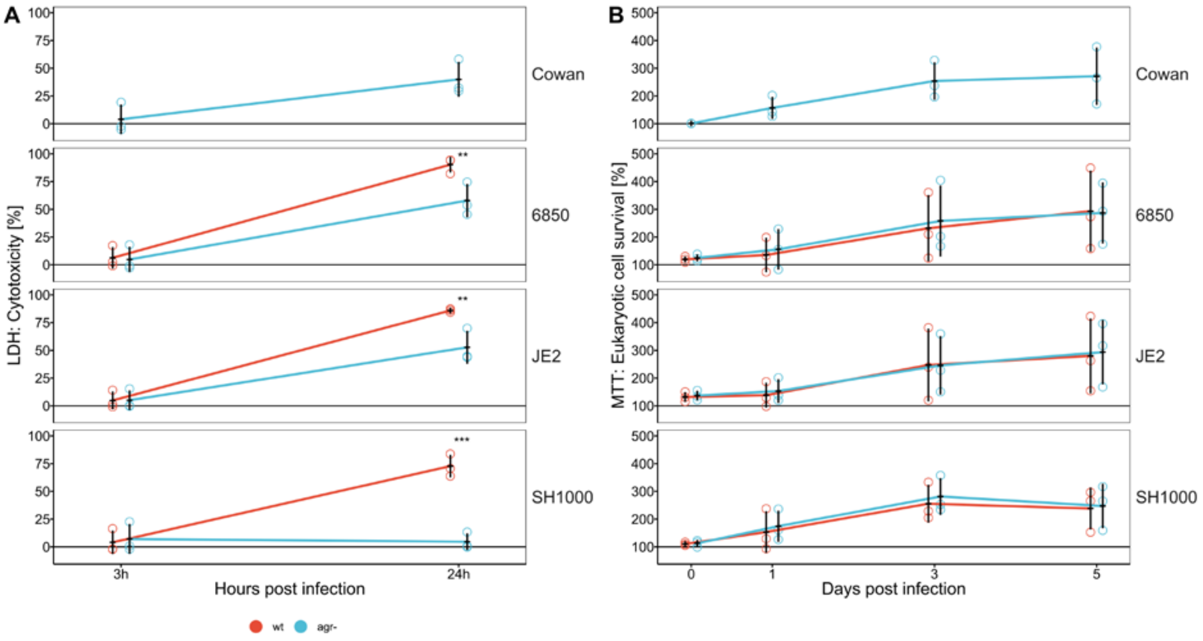

Supplementary Figure 2. Eukaryotic cell survival and cytotoxicity. **(A)** LDH release. Eukaryotic cell lysis was assessed 3 h and 24 h after infection. At the indicated time points, CytoTox 96® Reagent was mixed 1:1 with cell supernatant and absorbance was measured at 490 nm. Eukaryotic cell survival was normalized to a lysis control. Data points of wild types and *agr* mutants are offset on x-axis for easier readability. Pairwise comparison between 6850 wt and 6850 agr- ** p = 0.001, pairwise comparison between JE2 wt and JE2 agr- ** p = 0.0007 and pairwise comparison between SH1000 wt and SH1000 agr- *** p < 0.0001. **(B)** MTT assay. Eukaryotic cell survival was monitored over an infection period of five days. At the indicated time points, MTT solution was added to the cells. Formazan crystals formed by viable eukaryotic cells were dissolved and absorbance was measured at 570 nm. Increasing absorbance indicates increasing numbers of eukaryotic cells. Data points of wild types and *agr* mutants are offset on x-axis for easier readability.

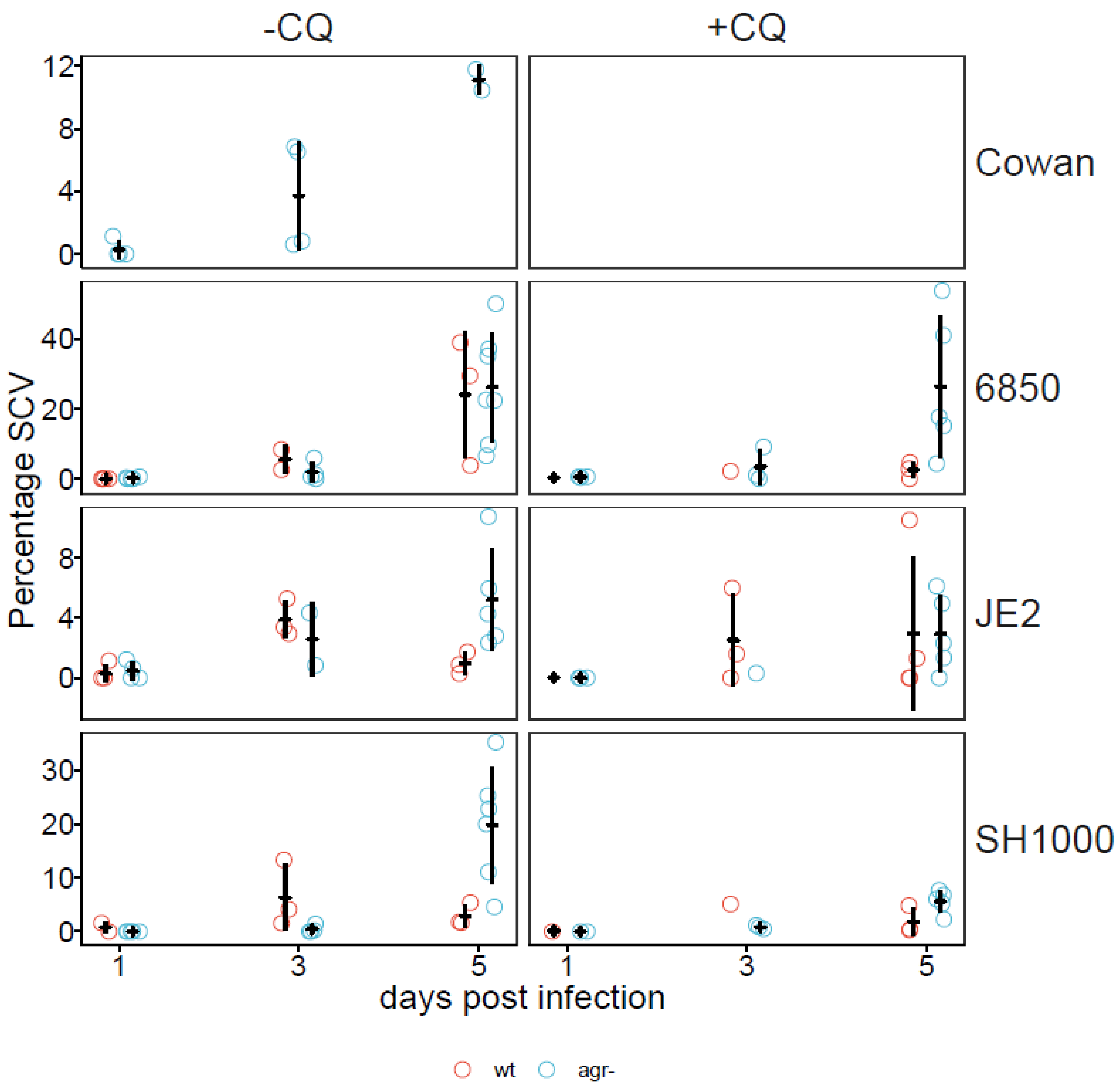

Supplementary Figure 3. Percentage SCVs. The threshold for SCVs was calculated as 1/5th of the area of the median area at day 0 for each strain. Each dot represents one biological repeat ± SEM. The alkalizing agent chloroquine (CQ) was used to neutralize the intracellular milieu, -CQ indicates no treatment, +CQ indicates chloroquine treatment. Data points of wild types and *agr* mutants are offset on x-axis for easier readability. One to seven independent experiments ± SEM are shown.

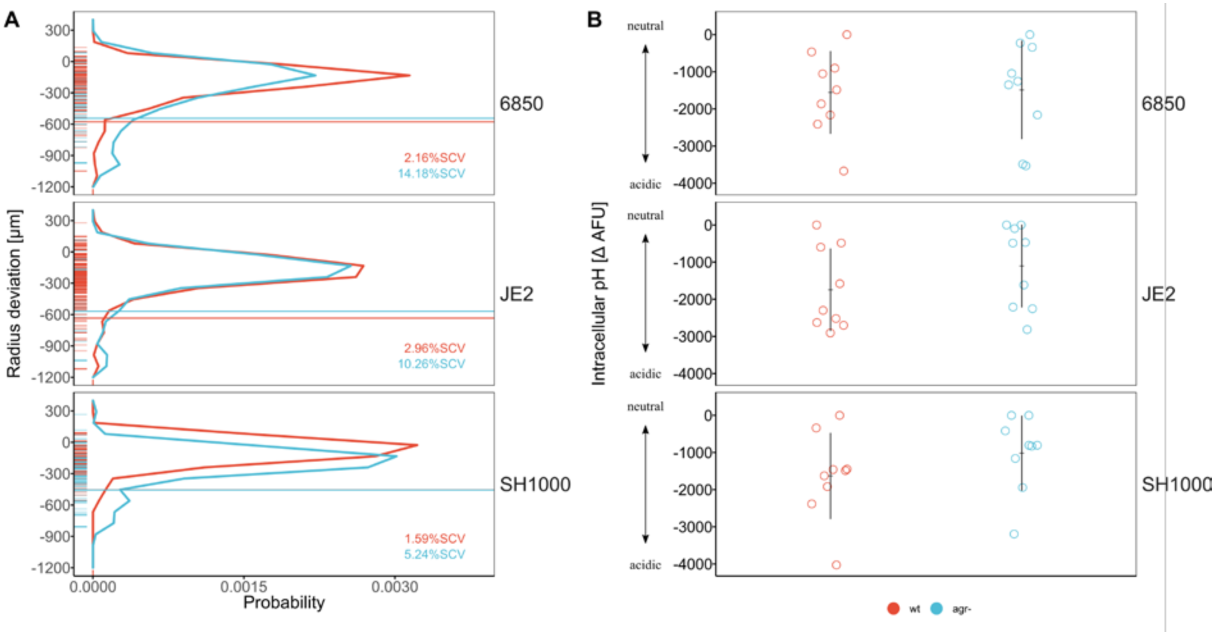

Supplementary Figure 4. Colony size distribution and exposure to intracellular pH of *S. aureus* wild type strains and *agr* mutants in the human skin fibroblast cell line BJ-5ta. *S. aureus* wild type strains are indicated in red, *agr* mutants are indicated in blue. **(A)** Colony size distribution of recovered bacteria. After 24h regrowth, colony radius is normalized to the strain specific mean colony area at day 0. Radius size is plotted as probability density function. Individual colonies are shown as short lines on y-axis. Long horizontal lines indicate the thresholds for SCVs calculated as 1/5th of the area of the median area for each strain. In total 1,887 colonies in three to four biological repeats were analyzed. The threshold for SCVs, indicated as a horizontal line, was calculated as 1/5th of the area of the median area at day 0. The mean percentage of SCVs is written in each panel. **(B)** Measurement of intracellular pH after five days of infection. Nine independent experiments in technical triplicates ± SEM are shown.

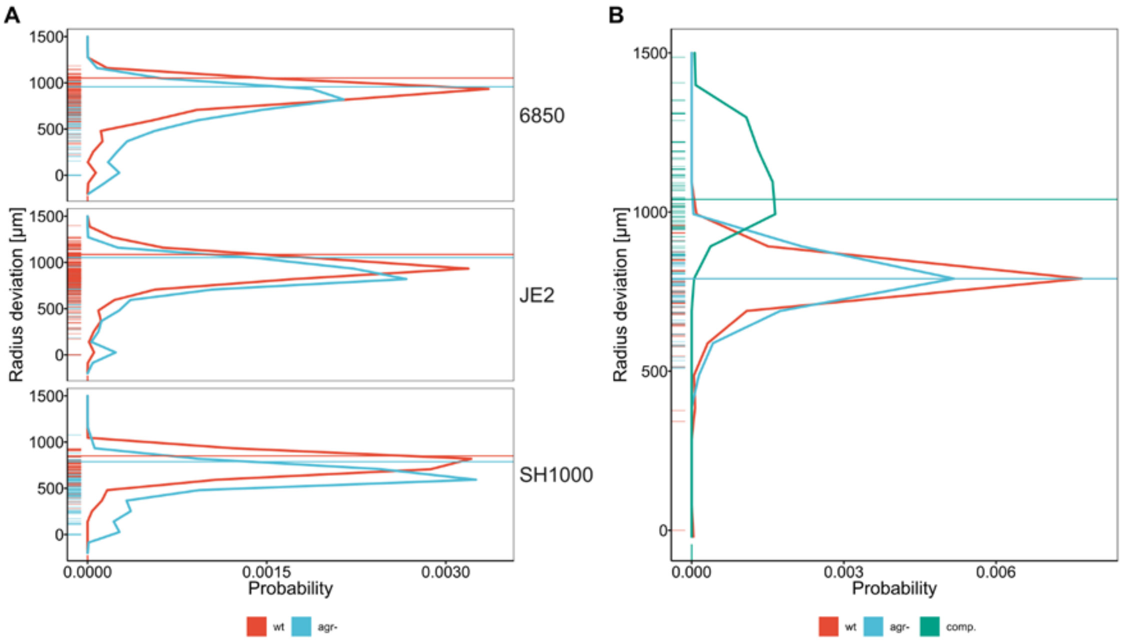

Supplementary Figure 5. Colony size distribution at day 0 of **(A)** *S. aureus* recovered from BJ-5ta cells and **(B)** *agr* complemented SH1000 recovered from A549 cells. *S. aureus* wild type strain is indicated in red, *agr* mutants are indicated in blue and *agr* complemented strain is indicated in green. After 24 h regrowth on blood agar plates, colony radius was measured. Radius size is plotted as probability density function. Individual colonies are shown as short lines on y-axis. Long horizontal lines indicate the thresholds for SCVs calculated as 1/5th of the area of the median area for each strain. In total 2,363 colonies were analyzed in two to four biological repeats.

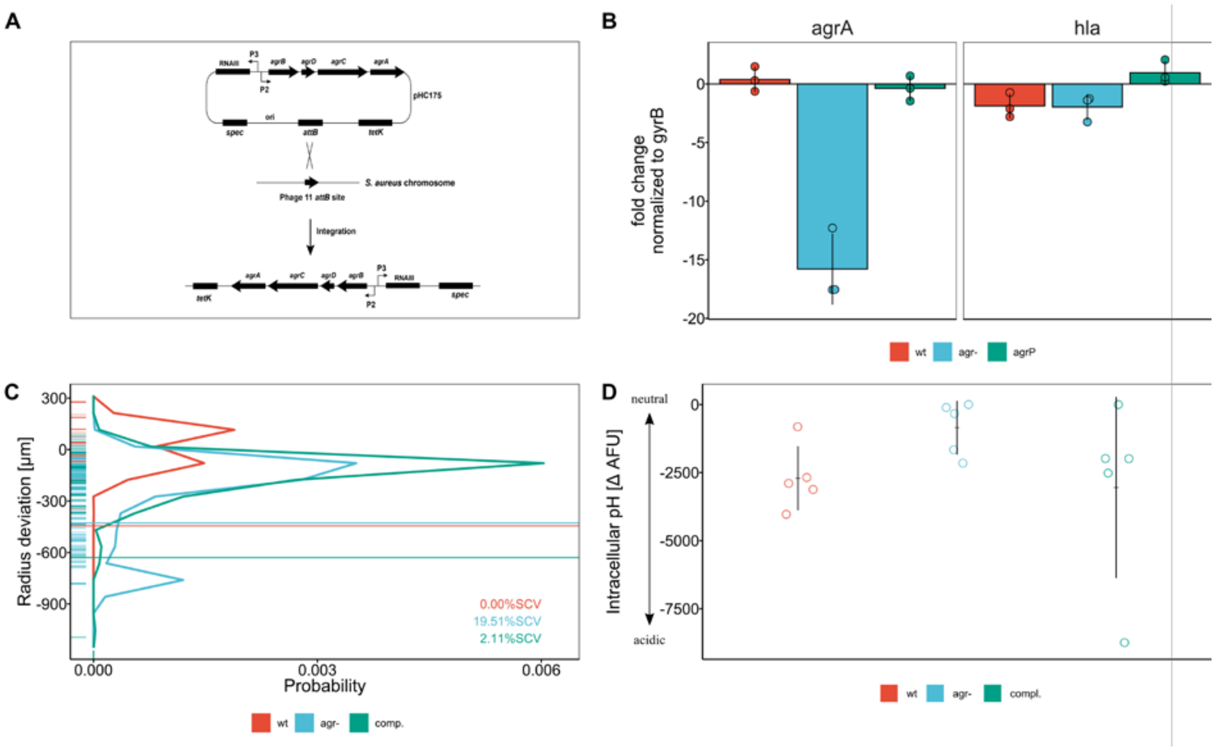

Supplementary Figure 6. Complementation of *agr* in *S. aureus* strain SH1000. *S. aureus* wild type strain is indicated in red, *agr* mutant is indicated in blue and *agr* complemented strain is indicated in green. **(A)** Construct for *agr* complementation. **(B)** Quantification of *hla* and *agrA* transcription using real-time PCR. Expression levels of *agrA* and *hla* was normalized to the housekeeping gene gyrase. Two independent experiments in technical triplicates ± SEM are shown in fold change. **(C)** Colony size distribution of recovered bacteria. After 24 h regrowth, colony radius is normalized to the strain specific mean colony area at day 0. Radius size is plotted as probability density function. Individual colonies are shown as short lines on y-axis. Long horizontal lines indicate the thresholds for SCVs calculated as 1/5th of the area of the median area for each strain. In total 1,022 colonies in two to six biological repeats were analyzed. **(D)** Measurement of intracellular pH after five days of infection. Five independent experiments in technical triplicates ± SEM are shown.

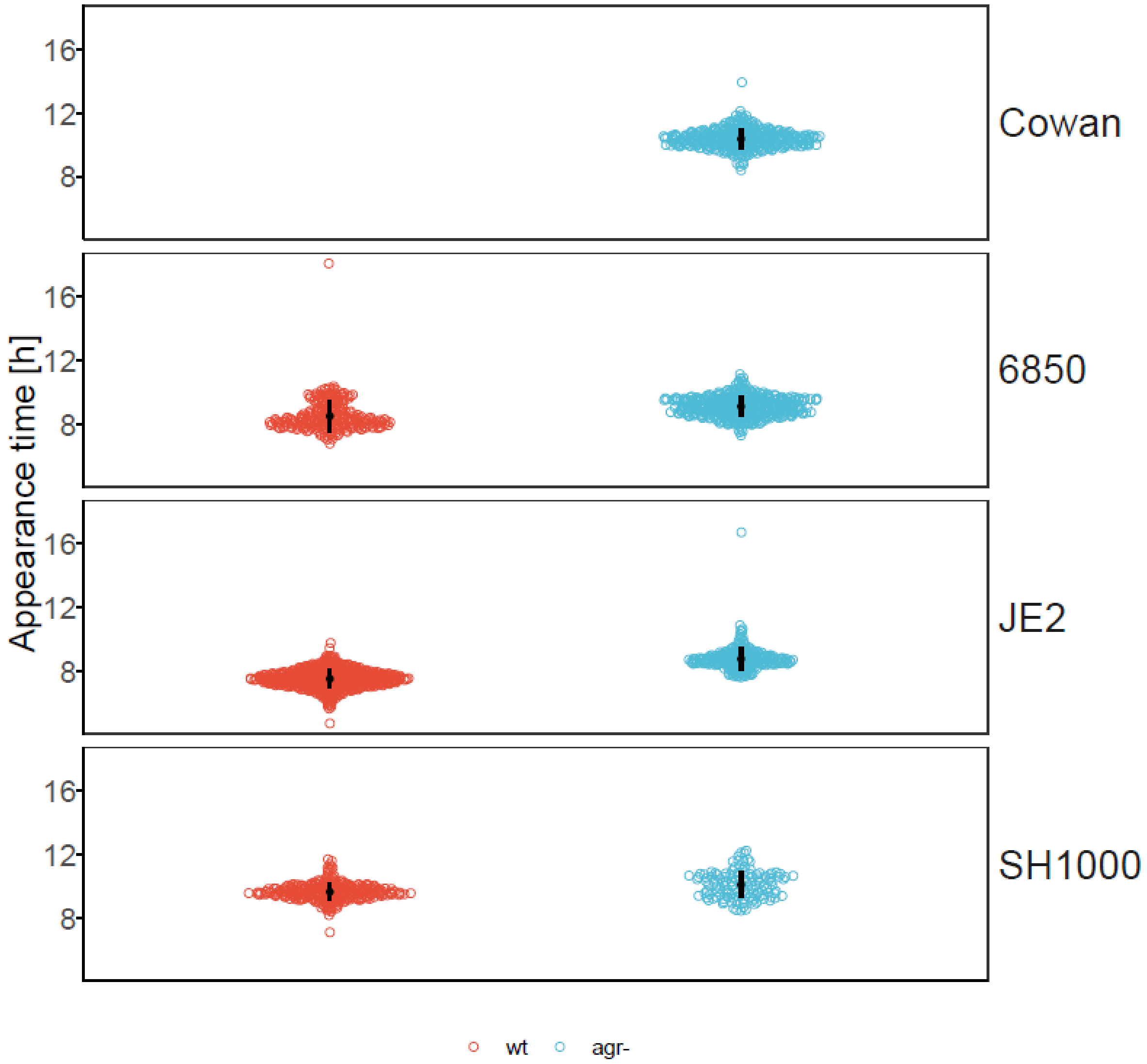

Supplementary Figure 7. Colony appearance time at day 0. Recovered bacteria were plated on blood agar and colony appearance was monitored by time-lapse analysis. Appearance time is plotted in hours. Every dot represents one colony ± SEM. For Cowan 354 colonies, for 6850 648 colonies, for JE2 850 colonies and for SH1000 415 colonies were analyzed in three biological repeats.

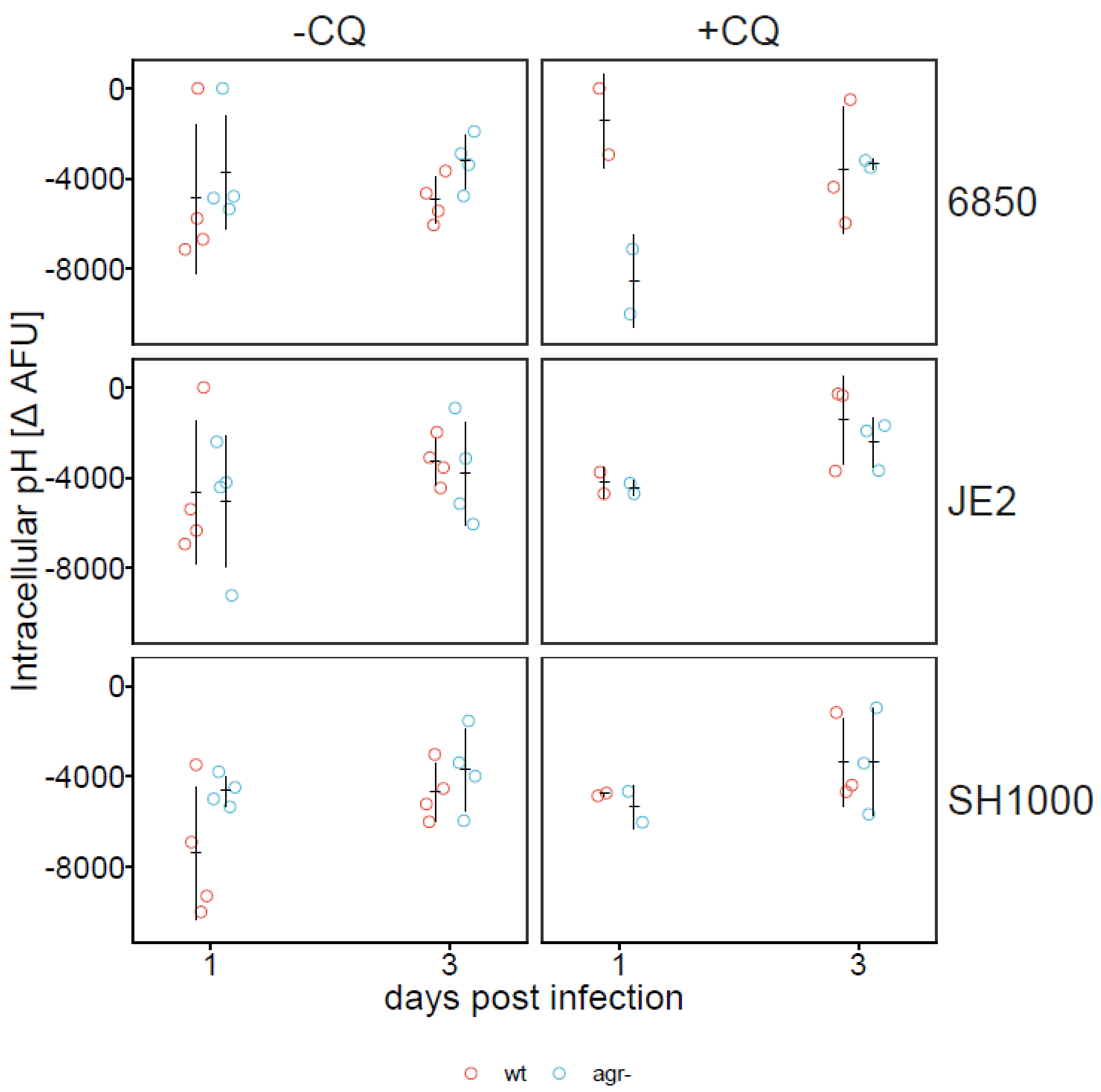

Supplementary Figure 8. Intracellular pH. Measurement of intracellular pH after 1, 3 and 5 days of infection. The alkalizing agent chloroquine (CQ) was used to neutralize the intracellular milieu. -CQ indicates no treatment, +CQ indicates chloroquine treatment. Larger negative ΔAFU values indicate a more acidic pH. Data points of wild types and *agr* mutants are offset on x-axis for easier readability. Two to ten independent experiments in technical triplicates ± SEM are shown.

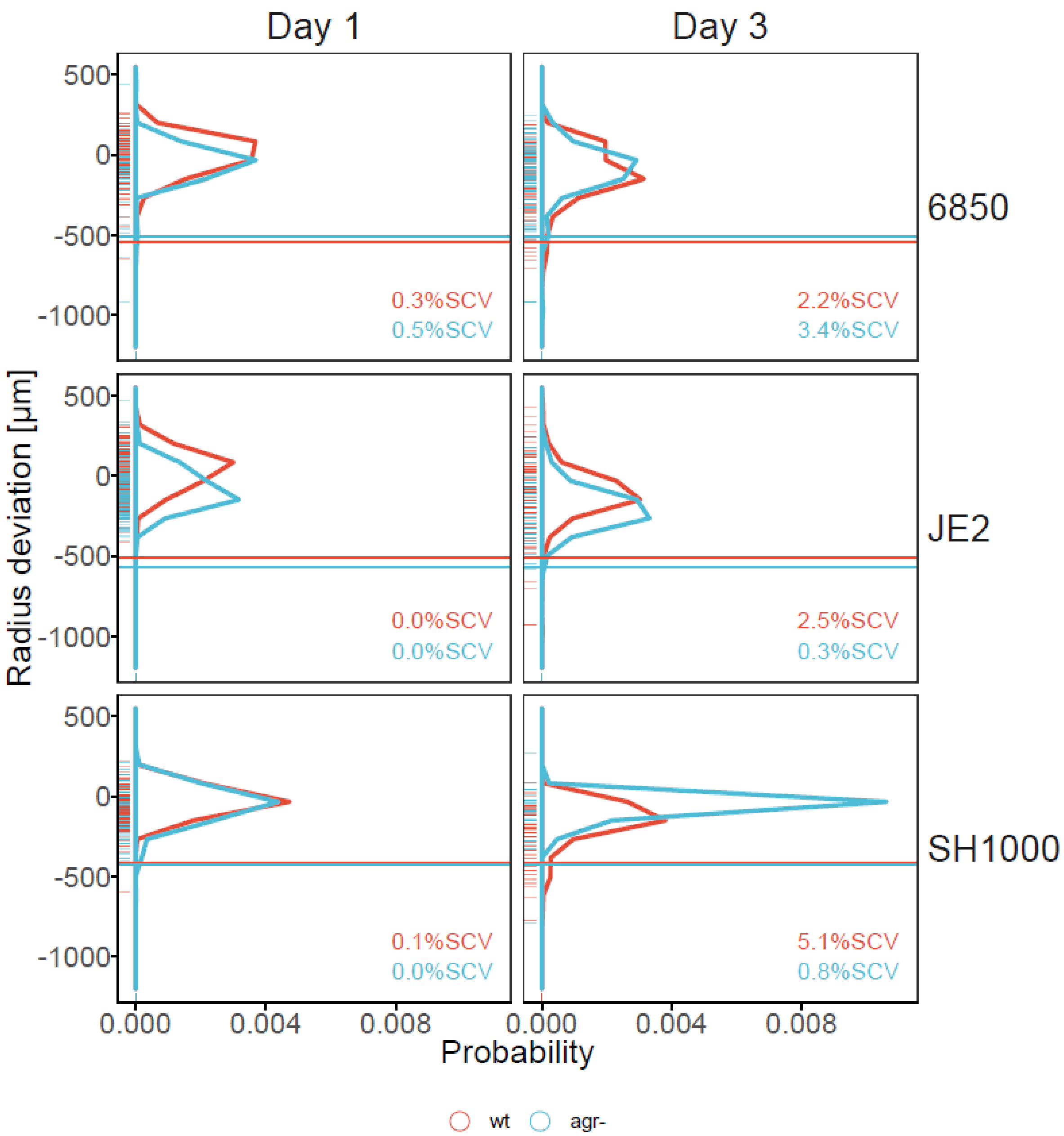

Supplementary Figure 9. Colony size distribution and SCV formation after 1 and 3 days of infection and chloroquine treatment. After 24 h regrowth on blood agar plates, colony radius was measured. Radius size is plotted as probability density function. Individual colonies are shown as short lines on y-axis. Long horizontal lines indicate the thresholds for SCVs calculated as 1/5th of the area of the median area for each strain. Colonies from single images as well as from time-lapse analysis were taken into account. For 6850 1,590 colonies, for JE2 2,349 colonies and for SH1000 1,823 colonies were analyzed in two to three biological repeats.
